# A facile, rapid procedure for Knoevenagel condensation reaction catalyzed by efficient amino-bifunctional frameworks under mild conditions

**DOI:** 10.1038/s41598-023-42832-5

**Published:** 2023-09-20

**Authors:** Mahdie Saghian, Saeed Dehghanpour, Zahra bayatani

**Affiliations:** Department of Inorganic Chemistry, Faculty of Chemistry, Alzahra University, P.O. Box 1993891176, Tehran, Iran

**Keywords:** Chemistry, Catalysis

## Abstract

A series of bifunctional hexagonal MOFs have been successfully constructed by the introduction of various amine functional groups within the unsaturated Cu-based MOF, HKUST, to access amino-modified frameworks. The prepared compounds are cost-effective and display high chemical and thermal stability. They were effectually exploited as efficacious and superb heterogeneous catalysts in rapid and facile Knoevenagel condensation reactions for a variety of substrates containing different electron-donating and electron-withdrawing substituents with very high conversion, good reusability under mild conditions, and very short reaction time. The contaminant presence of Lewis acid and basic sites resulted in efficient condensation reactions by the prepared catalysts.

## Introduction

Knoevenagel condensation is one of the most widely employed reactions in the industry which is used to produce imperative intermediates for pharmaceuticals, fine chemicals, functional polymers, and biologically active materials^1–3^. The fundamental condensation reactions took place between aromatic carbonyl compounds and activated methylene groups to construct carbon–carbon bonds^4–6^.

Various homogeneous catalysts were utilized in the condensation reactions such as ammonium salts, diverse amine groups and complexes, and organometallic catalysts^7–9^. However, homogeneous systems deficiencies and limitations such as catalyst recovery, difficult and costly product separation make researchers develop strategies to form efficient heterogeneous catalysts^10^. Therefore, different heterogeneous catalysts such as zeolites, mesoporous silica, metal oxides, and ionic liquids were ascertained to employ in condensation reactions^11^. Although developing appropriate catalysts is still a challenging issue for scientists.

For this purpose, porous crystalline compounds have attracted Scientific attention toward their research owing to their unique characterization as high surface area and well-ordered structures and porosity^12–14^. Among different categories of porous crystalline structure, the newly developed metal–organic frameworks (MOFs) which are constructed from metal ion clusters bridged by multidentate organic linkers, are exclusively exploited in various valuable applications such as catalysis, drug delivery, adsorption, separation and etc.^15–17^. These compounds are considered a new generation of catalysts with widespread research interest in recent decades due to their distinctive properties such as tunable pore size, high thermal stability, designable channels, high specific surface area, recoverability, tunable structures, etc. which make them exceptional heterogeneous catalysts^18–20^.

The presence of basic sites within the structure can promote and accelerate the Knoevenagel condensation reactions and is vital for reaction progress^21–23^. Amine functional groups are one of the good electrondonating groups that utilize in diverse reactions where basic sites are required^24–26^. Thus, MOFs with basic functional groups have been widely investigated for condensation reactions in recent years^27–29^. On the other hand, MOFs have organic ligands and unsaturated metal center sites which can undergo post-synthetic modification (PSM). Therefore, designing and applying ligands with appropriate functional groups is a crucial strategy for the fabrication of modified MOFs with novel structural characteristics and desired applications.

**Figure 1:**
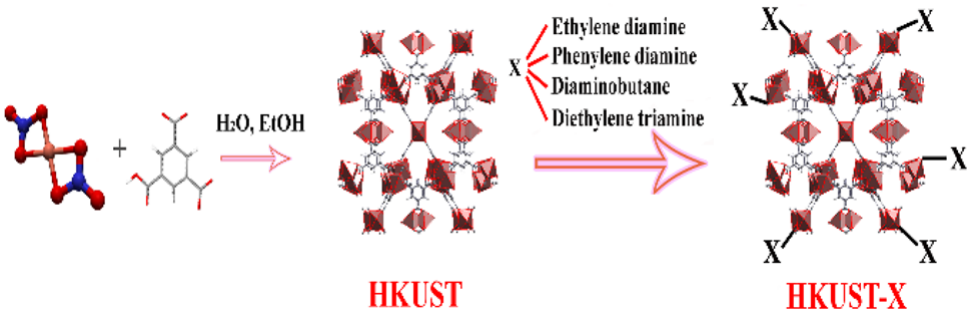
Schematic illustration of HKUST-X synthesis procedure.

With the above consideration in mind, in this work, HKUST was synthesized by solvothermal method and various linear amine functional groups were introduced into the unsaturated metal centers of the framework with refluxing method (Fig. 1). The prepared structures were utilized as bifunctional superb heterogeneous catalysts for the facile Knoevenagel condensation reactions between different benzaldehyde derivatives and malononitrile. The performance of the catalysts was compared with each other under mild reaction conditions.

## Experimental section

### Materials and instrumentation

Materials and instrumentation are explained in supporting information.

### Synthesis of catalysts

#### Synthesis of HKUST

According to the previously reported procedure, for synthesis, 3.6 mmol cupper (II) nitrate trihydrate (865 mg) and 2 mmol of trimesic acid (420 mg) were separately dissolved in 12 ml of deionized water and ethanol, respectively^30^. The obtained solutions were mixed together and stirred for 5 min at room temperature and transferred to a 50-ml Teflon-lined steel autoclave, heated at 120 °C for 24 h. The obtained blue powder was filtered by centrifuging and washed with a mixture of ethanol and deionized water in the same proportion and then activated by drying at 150 °C for 24 h.

#### Synthesis of HKUST-ED

In a typical procedure, 0.1 g of activated HKUST was suspended in 20 ml toluene following by the addition of 50 µl (0.7 mmol) ethylene diamine. The suspension refluxed for 16 h, cooled to room temperature, isolated by filtration. The resulting participate was washed with dichloromethane to eliminate unreacted ligands and dried at 100 °C for 3h.

#### Synthesis of HKUST-PhDA

This compound was synthesized under reflux conditions by treating HKUST and 1,2-phenylene diamine. A solution of 1,2-phenylene diamine (8o mg, 0.5 mmol) in 30 ml dichloromethane was prepared and added to a suspension containing 0.1 g of activated HKUST in 10 ml toluene. The reaction mixture was refluxed for 16 h and the resulting powder was collected by centrifuging, washed with dichloromethane, and dried at 100 °C for 3h.

#### Synthesis of HKUST-DA

The synthesis procedure for HKUST-DA is completely similar to HKUST-PhDA synthesis method. The difference is that 70 mg of 1,4-diaminobutane (0.8 mmol) is used instead of 1,2-phenylene diamine.

#### Synthesis of HKUST-DiT

In a typical procedure, 86 µl (0.8 mmol) of diethylene triamine was added to a suspension containing 0.1 g of activated HKUST in 30 ml. The light-blue powder formed was separated by filtration, washed with dichloromethane several times to purify. Therefore, the final product was dried at 100 °C for 3h.

### General procedure for Knoevenagel condensation reactions

To a 25 ml of round-bottomed flask containing 10 ml ethanol, malononitrile (66 mg, 1 mmol) was added. Then 10 mg of the prepared catalyst and 1mmol of benzaldehyde substrate was introduce to the aforementioned solution. The reaction mixture was stirred at room temperature for 5 min. The catalyst was isolated after the completion of the catalytic process and the ultimate filtered solution was characterized by gas chromatography.

## Results and discussion

### Characterization of catalysts

The FT-IR spectra of the pristine and modified structures was demonstrated in Fig. S1. As shown in the FT-IR spectrum of HKUST, the characteristic bands at 728 and 762 cm^−1^ were related to the out-of-plane deformation vibration of C‒H groups in the phenyl rings^31^. The emerged bands at 1378 and 1642 cm^−1^ can be attributed to the symmetric and asymmetric stretching vibration of carboxylate functional groups^32^. In addition, the broad stretching vibration band of the hydroxyl group of water molecules appeared at 3450 cm^−1^. The FT-IR spectra of HKUST-ED, HKUST-PhDA, HKUST-DA, and HKUST-DiT were represented in Figs. S1b, S1S1c, S1d, and S1e, respectively. The FT-IR spectra of functionalized structures are identical to the pristine MOF after incorporating diverse amine functional groups into the framework confirming successful modification of the framework. Moreover, the peak that is related to the N–H amine functional groups appeared at about 2700–3500 cm^−1^. On the other hand, it can be observed that the amount of amine functional groups loaded within the structure is very low (about 13–24%, Table S1). This low amount of loading causes the peaks related to N–H of amine group overlap with the broad band of the hydroxyl group of water molecules and eventually, the peaks related to N–H Amines cannot be seen well.

**Figure 2:**
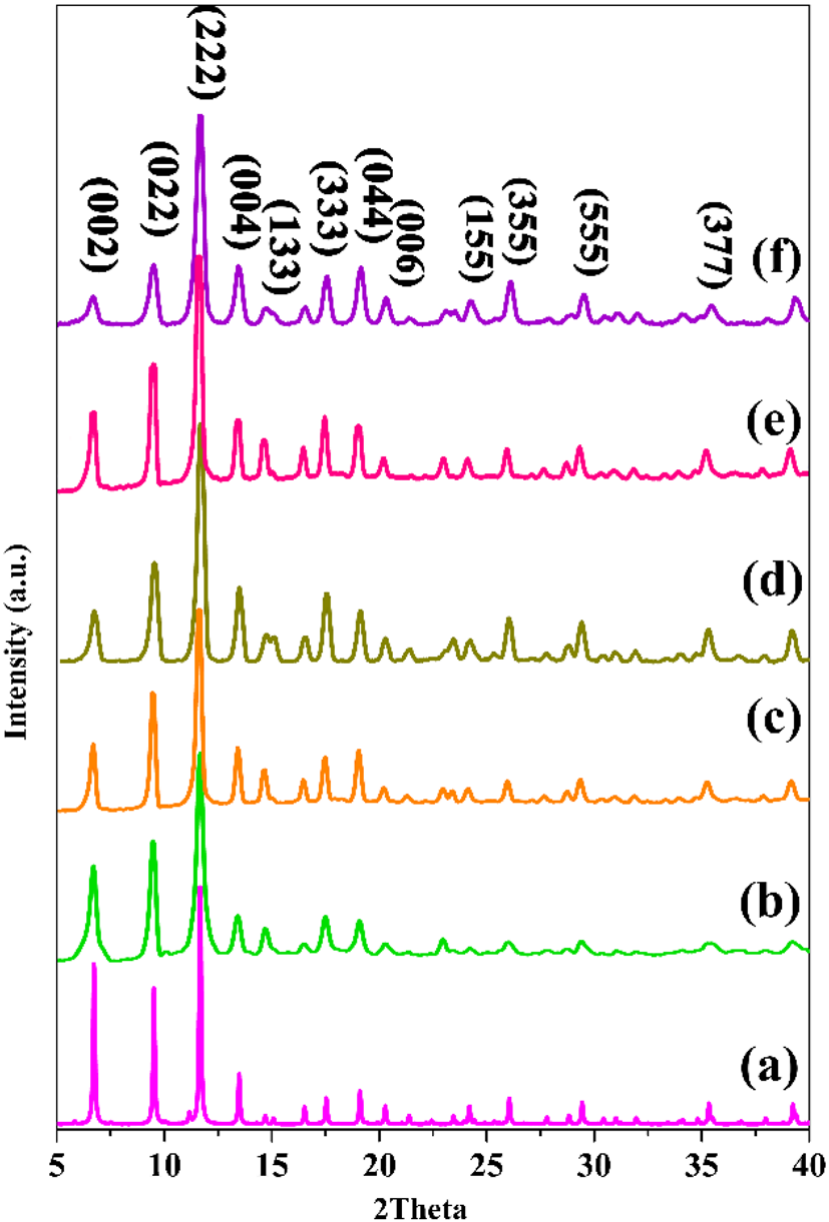
XRD patterns of HKUST (**a**) simulated (**b**) as-synthesized, (**c**) HKUST-ED, (**d**) HKUST-PhDA, (**e**) HKUST-DA, and (**f**) HKUST-DiT.

PXRD patterns of the as-synthesized and modified framework are presented in Fig. 2. XRD patterns of the pristine and functionalized framework corroborated well with the stimulated one and also the main peaks are well consistent with simulated data^30^. All prepared frameworks retained their crystallinity structures even after the introduction of amine functional groups into the frameworks demonstrating the chemical stability and structural integrity and robustness of the accumulated compounds. The XRD patterns of these samples, match well with CIF with CCDC 987,873.

**Figure 3:**
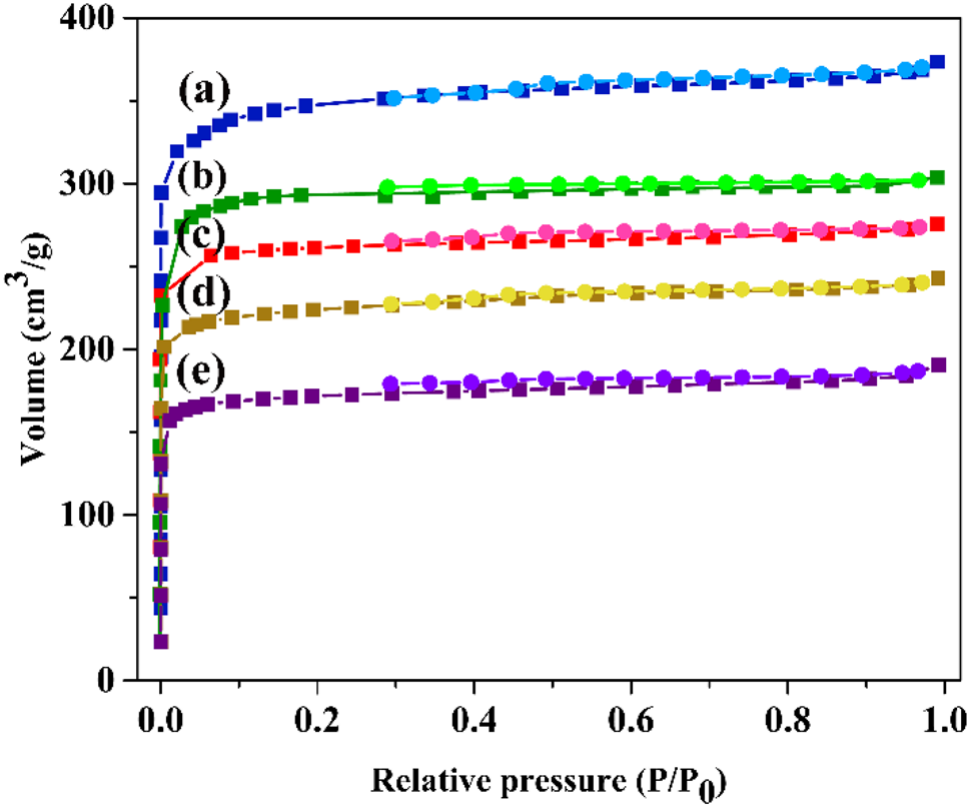
N_2_ adsorption–desorption isotherms of (**a**) HKUST, (**b**) HKUST-ED, (**c**) HKUDT-DA, (**d**) HKUST-DiT, and (**e**) HKUST-PhDA.

N_2_ adsorption–desorption isotherms were measured at low temperatures in order to identify the nature of porosity and surface area of the prepared frameworks. The textural parameters and N_2_ adsorption–desorption isotherms of the synthesized material are presented in Table 1 and Fig. 3, respectively. Based on the results, type I isotherms was revealed for both pristine HKUST and modified structures. Furthermore, the amount of surface area and pore volume for HKUST is 1363 m^2^/g and 0.58 cm^3^/g, respectively. After the introduction of amine functional groups into the framework, the relevant amount was decreased to 983 m^2^/g and 0.46 cm^3^/g, 929 m^2^/g and 0.42 cm^3^/g, 894 m^2^/g and 0.37 cm^3^/g, and 683 m^2^/g and 0.31 cm^3^/g for HKUST-ED, HKUST-DA, HKUST-DiT, and HKUST- PhDA, respectively. The decrement in surface area and pore volume amount affirmed the successful incorporation and modification of frameworks.

**Table 1:** Textural parameters of HKUST and amino-functionalized of HKUST.

Entry	Sample	S_BET_ (m^2^ g^−1^)	Pore volume (cm^3^ g^−1^)
1	HKUST	1363	0.58
2	HKUST-ED	983	0.46
3	HKUST-DA	929	0.42
4	HKUST-DiT	894	0.37
5	HKUST- PhDA	683	0.31

**Figure 4:**
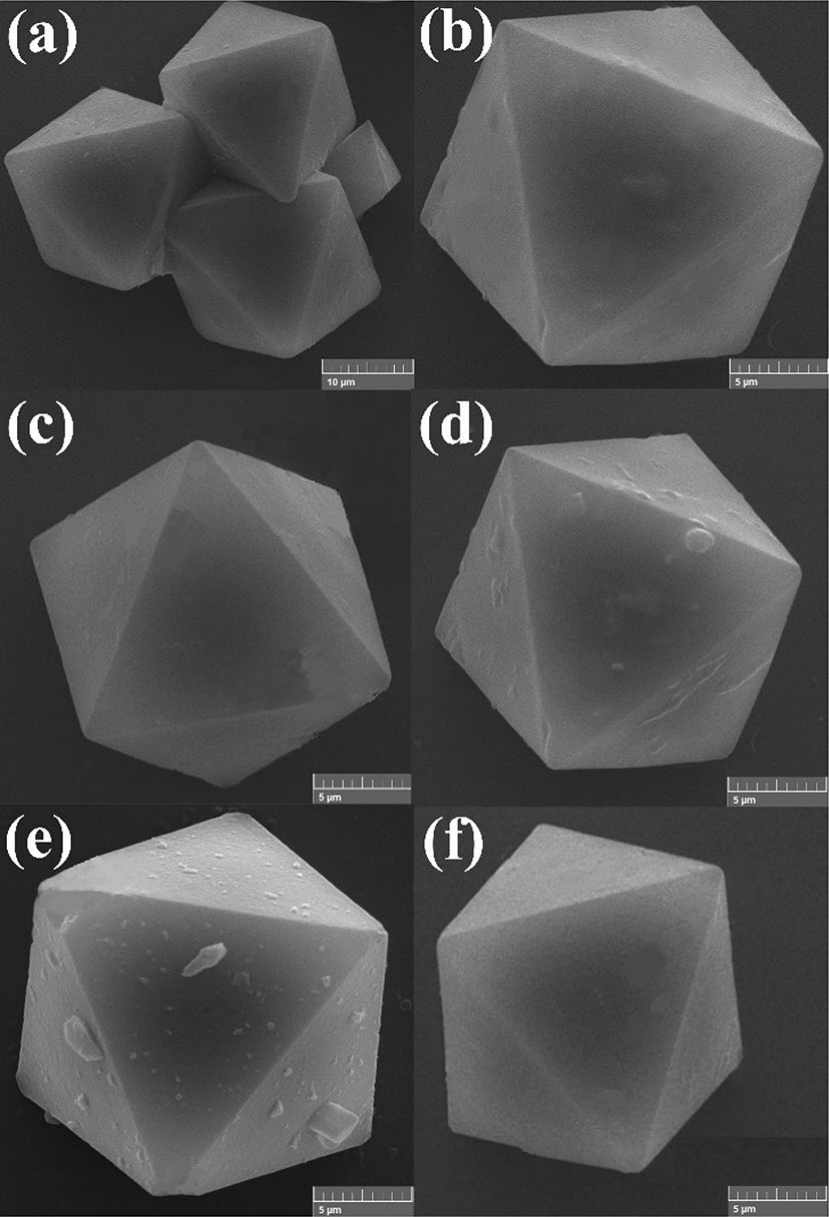
SEM images of (**a**), (**b**) HKUST, (**c**) HKUST-ED, (**d**) HKUDT-PhDA, (**e**) HKUST-DA, and (**f**) HKUST-DiT.

In order to evaluate the morphology of the prepared compounds, scanning electron micrograph analysis was carried out and the result micrographs are presented in Fig. 4. According to the obtained SEM images, the morphology of HKUST is characterized by a well-defined octahedral structure (Fig. 4, b). The morphology of structures remained unchanged after modification affirming the successful introduction of desired groups into the structures (Fig. 4c-f). Moreover, TEM image of one of the modified structures (HKUST-ED) are depicted in Fig. S2.

TGA analysis was accomplished in order to investigate the thermal behavior and stability of the prepared frameworks and the results are shown in Fig. S3. Accordingly, for HKUST and functionalized frameworks, two weight losses were observed. The first weight loss in the TG curve of HKUST at about 70–120 °C belonged to the removal of coordinated and guest H_2_O molecules in the structure. The next weight loss that was perceived with the temperature increasing at about 350 °C, corresponds to structural collapse and degradation. Similarly, the functionalized frameworks have thermal stability up to 350 °C. Thus, the prepared frameworks demonstrate high thermal stability behavior.

The amount of amine functional groups loaded within the structure was acquired by elemental analysis and the results are summarized in Table S1. Based on the results, the amount of amine groups loaded into the framework was obtained due to the afforded experimental nitrogen percent. Therefore, the amount of loading for HKUST-ED, HKUST-PhDA, HKUST-DA, and HKUST-DiT were 13.5, 24.1, 18.3, and 21.7%, respectively.

With the above consideration in mind and according to all obtained results, amine functional groups were incorporated into the structure through the coordination of nitrogen atoms to the unsaturated metal centers in the framework^33–35^.

### Catalytic activity of the prepared frameworks

At first, efforts were made to synthesize HKUST, and then the incorporation of various amine functional groups was carried out into the framework. For functionalization, ethylene diamine, 1,3-phenylene diamine, 1,4-diaminobutane, and diethylene triamine were utilized in order to enhance the catalytic activity. The obtained frameworks named HKUST-ED, HKUST-PhDA, HKUST-DA, and HKUST-DiT and are also considered catalysts **1**, **2**, **3**, and **4**, respectively. The prepared catalysts were used for facile and rapid Knoevenagel condensation reactions under mild conditions to form an α, β-unsaturated ketones.

To figure out the role of amine functional group in condensation reactions, some experimental reactions were performed and the results are summarized in Table 2. First, the reaction was accomplished without the addition of any catalyst in the reaction media, and no conversion was observed for this reaction (Table 2, Entry 1). Second, the condensation reaction was performed in the presence of the pristine MOF (HKUST), and no substantial conversion was perceived in this case (Table 2. Entry 2). Next, in the case of utilization of either the physical mixture of amine groups with HKUST or the sole amine functional group, the reaction cannot proceed with a remarkable conversion (Table 2, Entries 3, 4, respectively). However, in the case of the physical mixture slight progress can be observed, but sequestration is a major problem in this case. In the case of using the prepared catalyst, complete progress of the reaction is observed and the conversion rate of 100% was obtained (Table 2, Entry 5).

**Table 2:** Catalytic activity of diverse catalysts^a^.

Entry	Catalyst	Conv (%)
1	Blank	–
2	HKUST	13
3	Ethylenediamine	5
4	HKUST/ED	19
5	HKUST-ED	100

^a^Reaction conditions: Benzaldehyde (1 mmol), malononitrile (1 mmol), Catalyst (10 mg), Room temperature, time (5 min).

### Knoevenagel condensation reactions

The catalytic activity of prepared catalysts (1, 2, 3, and 4) was investigated in Knoevenagel condensation reactions in terms of activity, selectivity, and reusability. For this purpose, benzaldehyde and malononitrile were chosen as substrate models in order to perform Knoevenagel condensation reactions. Different parameters such as time, amount of catalyst, temperature, and solvent were optimized and the results are displayed in Figs S4-7, respectively. According to the results, the condensation reaction was carried out within 5 min and at ambient temperature, in the presence of 10 mg of catalyst with an excellent conversion rate of 100%. In addition, Ethanol was designed as the optimized solvent of the reaction. Based on the results, protic solvents act better than aprotic solvents due to their polarity and the ability to stabilize intermediates (Fig. S7). Both ethanol and acetonitrile demonstrate high conversion for condensation reaction, but ethanol was elected due to its lower toxicity.

The obtained superb results led to examine different aromatic aldehydes with either electron-donating or electron-withdrawing functional groups in condensation reaction and the results are summarized in Table 3. As the results demonstrate, in a very short reaction time, all substrate converts to their related product (α, β- unsaturated ketone) with high conversion rates. It is worth noting that in these reactions the selectivity is 100% and α, β-unsaturated ketone is the sole product of the reaction. The type of substituent has a significant effect on the reaction efficacy. According to the results, benzaldehydes with electron-withdrawing functional groups act better than benzaldehydes with electron-donating groups, and higher conversion was obtained for them (Table 3, entries 2–6). Among various applied electron-withdrawing substitutes including nitro, chlorine, and bromine functional groups, the trend of catalytic performance enhanced as the following process; NO_2_ > Cl > Br. Also, the presence of substitution in the ortho position compared to the para position causes more activity of the catalyst (Table 3, entries 3,4). On the other hand, between diverse electron-donating benzaldehydes utilized in Knoevenagel condensation reactions containing methoxy and methyl groups, the increase in the catalytic performance of the substituents is as follows; OCH_3_ > CH_3_ (Table 3, entries 7–11). In the case of using hydroxybenzaldehyde, no catalytic activity and reaction progress was observed (Table 3, entry 12). Furthermore, when using 2,6- dichlorobenzaldehyde and 3,4- dimethoxybenzaldehyde as substrates, the catalytic activity reduced compared to the mono-substitute owing to the increased steric hindrance (Table 3, entries 5,9).

**Table 3:**
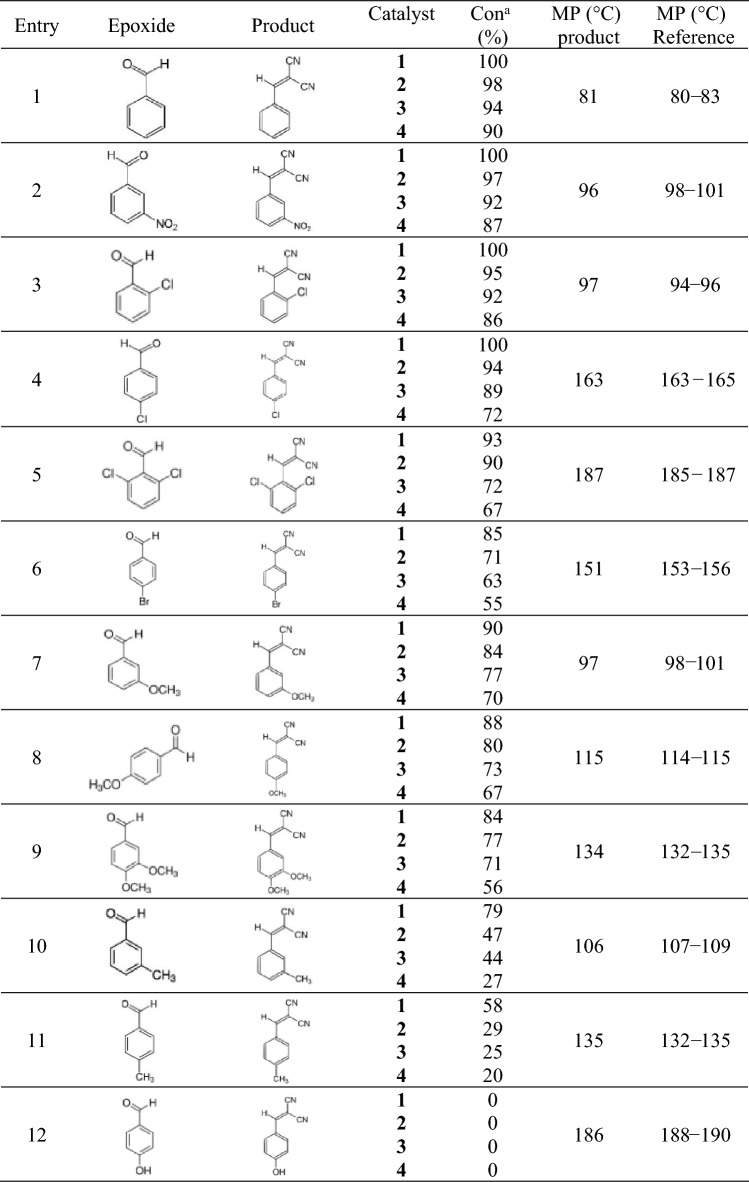
Results of Knoevenagel condensation reactions in the presence of used catalyst.

^a^Reaction conditions: Benzaldehyde (1 mmol), malononitrile (1 mmol), Catalyst (10 mg), Room temperature, time (5 min).

For further conformation, ^13^CNMR, ^1^HNMR, and FT-IR analysis were carried out for some products and the results were displayed in Figs S8-S6 which confirmed the formation of products.

Moreover, the catalytic activity of catalyst **1** was examined in Knoevenagel condensation reaction of benzyl alcohol with different contents of ethylene diamine loaded into the framework, and the results were represented in Table S2. As results shown, by decreasing the amount of loaded ethylene diamine, the catalytic performance of **1** was also reduced and less catalytic activity is observed.

### The plausible mechanism for Knoevenagel condensation reaction

**Figure 5:**
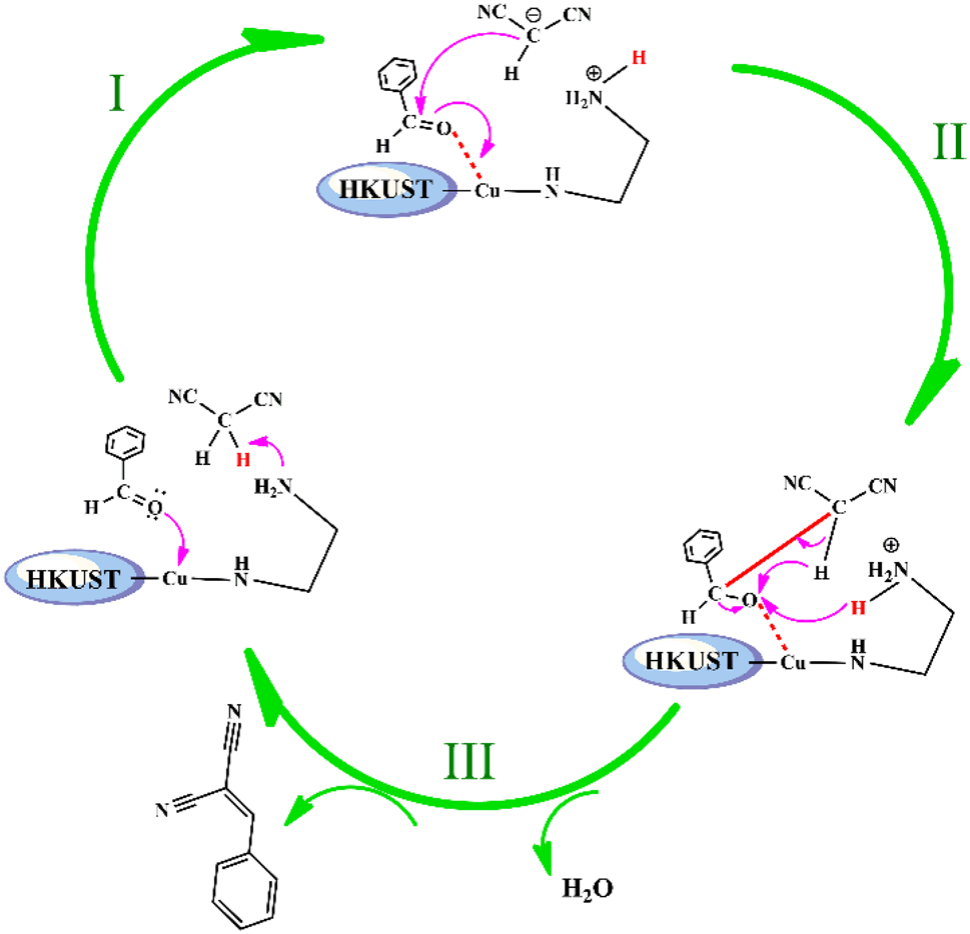
The plausible mechanism for Knoevenagel condensation reaction in the presence of HKUST-ED.

The proposed mechanism for Knoevenagel condensation reaction of benzaldehyde with malononitrile in the presence of catalyst **1** was illustrated in Fig. 5. On the basis of the proposed mechanism, metal centers and incorporated amine groups within the framework act as Lewis acid and basic sites, respectively. In the first step, carbonyl groups on benzaldehyde were activated by the metal ions of MOF, and simultaneously basic sites in the framework deprotonated malononitrile and formed a carbanionic intermediate (Fig. 5, pathway I). Subsequently, the next step continues with the attack of the carbanionic anion on the polarized carbonyl group and a new intermediate was generated (Fig. 5, pathway II). Ultimately, the final product was constituted with the elimination of H_2_O molecules and the catalyst was regenerated to participate in the next cycle at the same time (Fig. 5, pathway III). Therefore, based on the proposed mechanism, the presence of basic sites is indispensable for the reaction progress^36, 37^.

### Comparison of the catalytic performance of the synthesized compounds

Knoevenagel condensation reactions were performed in the presence of catalysts with various amine functional groups. The trend of catalytic performance of utilized catalysts is as the following process; **1** > **2** > **3** > **4**. Based on the aforementioned trend, catalyst **1** with ethylenediamine functional group exhibited the best results due to its proper orientation, availability of corresponding electron pairs, and free rotation of amine groups. In the case of using phenylene diamine as basic site in the framework (catalyst **2**), the electron pairs of the amine group are involved in resonance with the aromatic ring. With this regard, despite the proper orientation, a weaker performance was observed compared to ethylenediamine functional groups. In the case of using catalyst **3**, the farther distance of amine groups from metal ion centers, and sterically hindrance increment resulted in the worse performance of the catalyst and lower results were observed. Catalyst **4** is the least efficacious catalyst among the utilized catalysts. Although this catalyst has more basic sites, it exhibits weaker activity compared to others due to increased steric hindrance.

In order to investigate the catalytic performance of the synthesized frameworks, a comparison was made between the prepared catalyst and other previously reported heterogeneous catalysts based on MOFs applied in Knoevenagel condensation reaction. The mentioned compounds are compared in terms of activity with each other and the results are listed in Table 4. Based on the results, our synthesized catalyst revealed a substantial increment in catalytic activity. Furthermore, other advantages of the prepared catalysts include simplicity, effectiveness, very short reaction time, and accomplishing the reaction at room temperature.

**Table 4:** The comparison of catalytic activity of the present work with some previously reported catalysts for the Knoevenagel condensation reactions between benzaldehyde and malononitrile.

Entry	Catalyst	solvent	Time (min)	Temperature (°C)	Conversion (%)	Ref
1	UIO-66-NH-RNH_2_	THF	300	RT	84	^38^
2	**1**-Pd	DMSO	5	RT	42.5	^39^
3	ZIF-8	Toluene	360	RT	97	^21^
4	FeBTC	Xylene	360	130	93	^40^
5	CAU-1-NH_2_	Ethanol	420	40	91	^41^
6	Fe-MIL-101-NH_2_	Toluene	180	80	90	^42^
7	Zn-MOF	DCM	180	RT	80	^43^
8	Mn-MOF-Pi	DMF	180	RT	65	^44^
9	Fe_3_O_4_@ZIF-8	Toluene	180	RT	94	^45^
10	Au@Cu-MOF	Toluene	420	RT	99	^13^
11	TMU-5	Methanol	30	RT	68	^46^
12	Ni induced ZIF-8 nanoframes	Toluene	120	RT	68.8	^47^
13	Ni-MOF	*p*-Xylene	360	130	78	^48^
14	UPC-30	DCM	300	RT	94	^49^
15	Present work	Ethanol	5	RT	100	This work

The recyclability of the prepared catalysts was carried out via consecutive Knoevenagel condensation reactions under the optimized reaction conditions and the results are presented in Figs. S17-19. According to the results in Fig. S17, the catalyst can be recycled for five sequential runs without considerable loss of its activity. Moreover, the PXRD pattern and FT-IR spectra which depicted in Figs. S18 and S19, respectively revealed that the catalyst retains its crystallinity and structural integrity.

## Conclusion

In summary, an unsaturated Cu-based MOF, HKUST, was constructed and successfully modified with diverse amine functional groups. The prepared heterogeneous catalysts were applied as efficient catalysts for rapid and facile Knoevenagel condensation reactions. The Knoevenagel products were achieved with high conversion under mild conditions and very short reaction time compared to other previously reported catalysts. Other unique characteristics of the prepared catalysts include recyclability, the cost-effectiveness of the synthesized catalysts, and performing reactions at room temperature.

### Supplementary Information

Supplementary Information.

## Data Availability

The datasets used and/or analyzed during the current study available from the corresponding author on reasonable request.
