# Supplementary material for: A facile, rapid procedure for Knoevenagel condensation reaction catalyzed by efficient amino-bifunctional frameworks under mild conditions
Source: Sci Rep. 2023 Sep 20;13:15563. doi: 10.1038/s41598-023-42832-5 (PMC10511422; doi:10.1038/s41598-023-42832-5)
Supplement: Supplementary file 1 — Supplementary Information. [file 41598_2023_42832_MOESM1_ESM.pdf]

Supporting information:

Material and instrumentation

All reagents were purchased from Sigma-Aldrich and Merck chemical companies and used without further purification. FT-IR spectra were recorded on a Bruker Tensor 27 FTIR spectrophotometer using KBr pellets over the range of 4000–400 cm^{-1}. The X-ray powder diffraction (XRD) data were recorded on a Siefert XRD 3003 PTS diffractometer, using Cu Kα1 radiation (k = 1.5406 Å. Scanning electron microscopy (SEM) images were obtained on a Philips XL-30ESEM equipped with an X-ray energy dispersive detector. Transmission electron microscopy (TEM) analysis was performed by a Zeiss EM900 electron microscope. Thermogravimetric analysis (TGA) was performed using a Mettler Toledo TGA/DSC instrument with heating rate of 10 °C/min in an air atmosphere. Nitrogen sorption isotherms were recorded on a Belsorp Mini–II instrument at 77K. The ^{1}H NMR and ^{13}C NMR spectra were recorded on a Bruker 400 MHz and 125 MHz spectrometer respectively, using DMSO as solvent. Catalysis products were analyzed by GC and GC-Mass using Agilent 6890 series with a FID detector, HP-5, 5% phenylmethylsiloxane capillary and Agilent 5973 network, mass selective detector, HP-5 MS 6989 network GC system, respectively**.** Carbon dioxide sorption isotherms were recorded on a Brunauer-Emmett- Teller surface area and porosity analyzer at 298K.

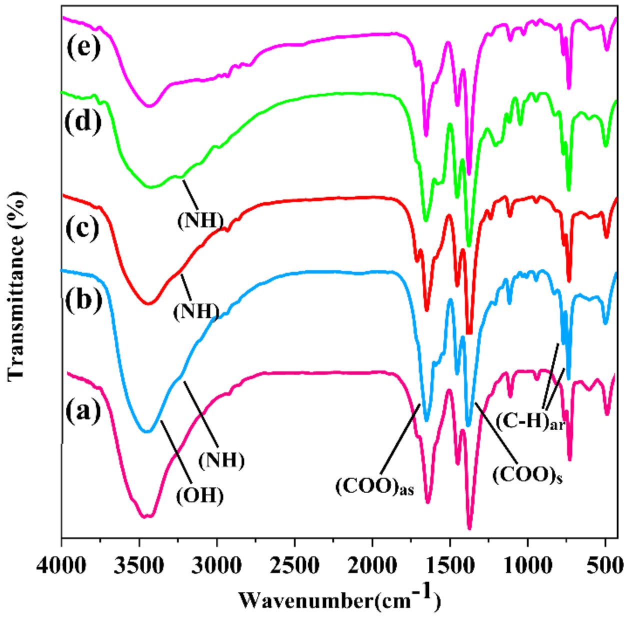

Fig. S1. FT-IR spectra of (a) HKUST, (b) HKUST-ED, (c) HKST-PhDA, (d) HKUST-DA, and (e) HKUST-DiT.

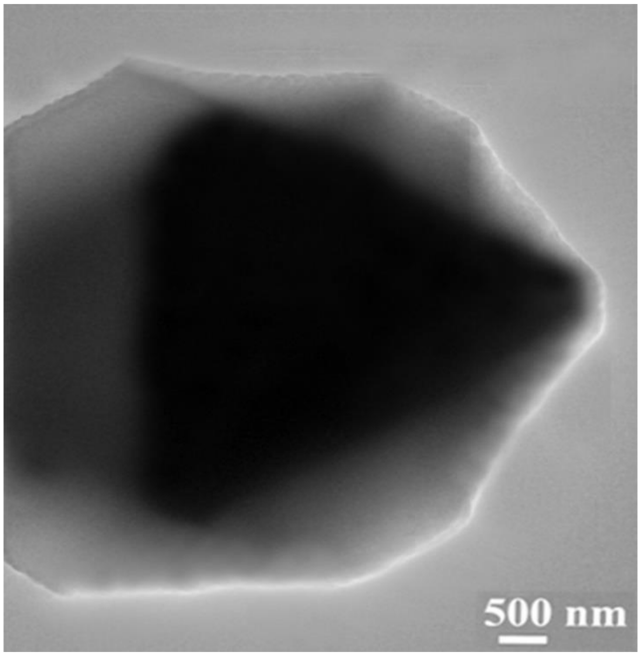

Fig. S2. TEM image of HKUST-ED.

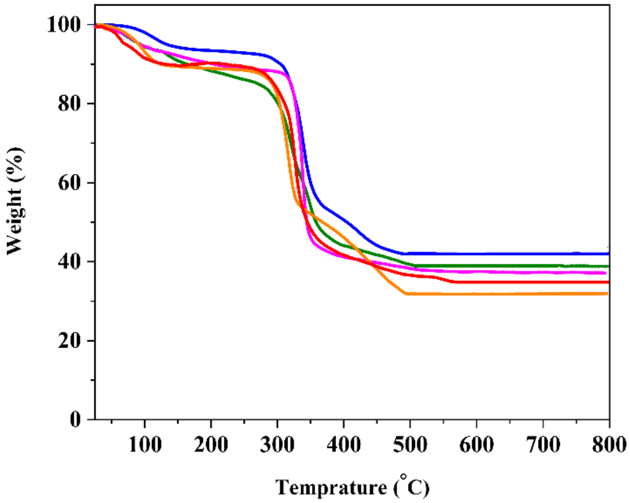

Fig. S3. TGA curves of (blue) HKUST, (green) HKUST-ED, (pink) HKST-DA, (red) HKUST-DiT, and (orange) HKUST-PhDA.

Table S1. Elemental analysis of amino-functionalized of HKUST.

Entry	Sample	C (%)	H (%)	N (%)	Amount of loading (%)
1	HKUST-ED	29.85	4.05	6.27	13.45
2	HKUST-PhDA	35.84	2.93	6.23	24.05
3	HKUST-DA	32.67	3.79	5.82	18.32
4	HKST-DiT	42.27	4.49	8.83	21.68

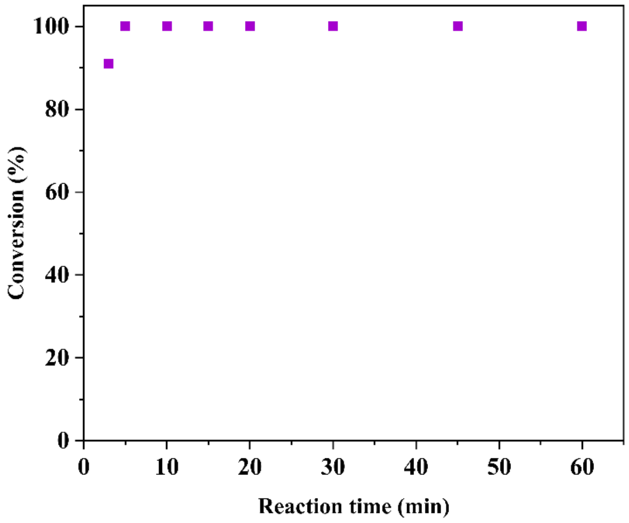

Fig. S4. The effect of reaction time on the Knoevenagel condensation of benzaldehyde with malononitrile over HKUST-ED.

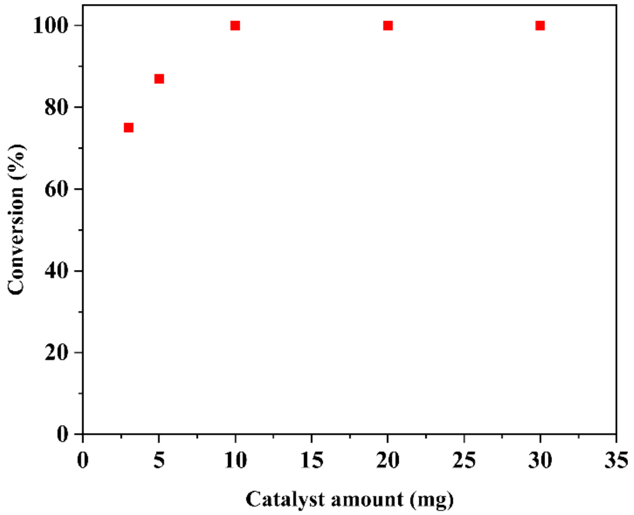

Fig. S5. The effect of catalyst amount on the Knoevenagel condensation of benzaldehyde with malononitrile over HKUST-ED.

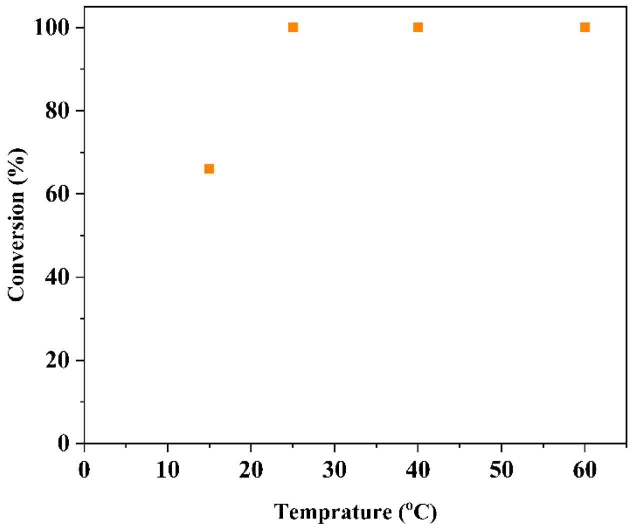

Fig. S6. The effect of temperature on the Knoevenagel condensation of benzaldehyde with malononitrile over HKUST-ED.

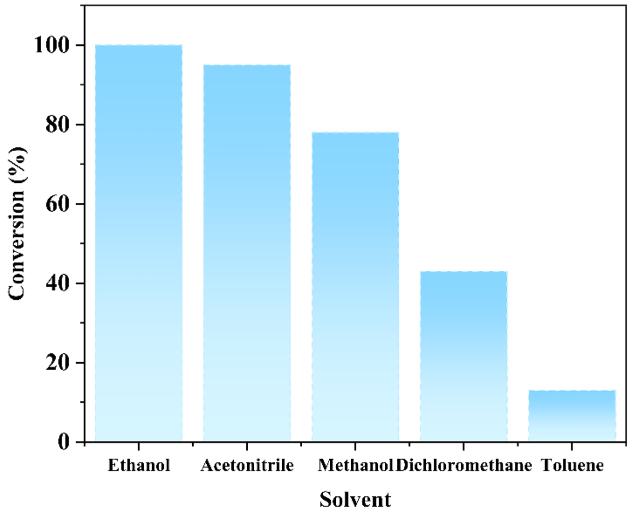

Fig. S7. The effect of solvent on the Knoevenagel condensation of benzaldehyde with malononitrile over HKUST-ED.

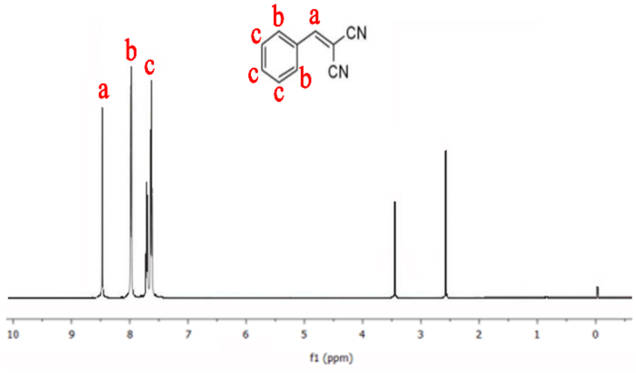

Fig. S8. ^{1}H NMR spectra of benzylidene malononitrile.

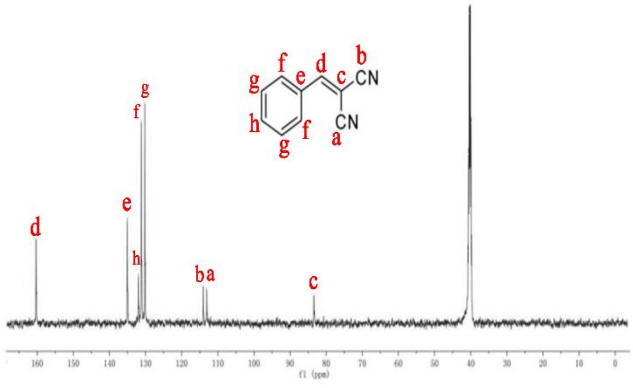

Fig. S9. ^{13}C NMR spectra of benzylidene malononitrile.

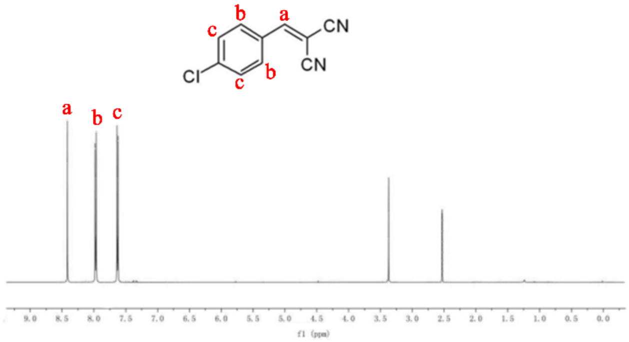

Fig. S10. ^{1}H NMR spectra of (4-chlorobenzylidene) malononitrile.

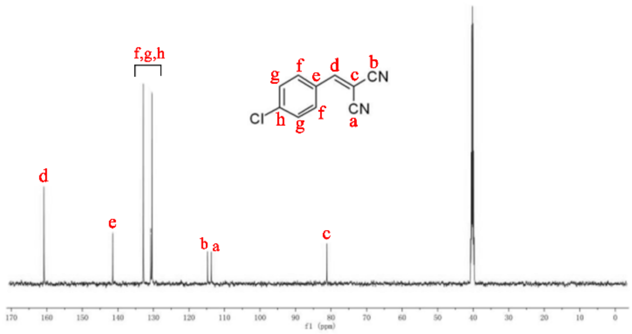

Fig. S11. ^{13}C NMR spectra of (4-chlorobenzylidene) malononitrile.

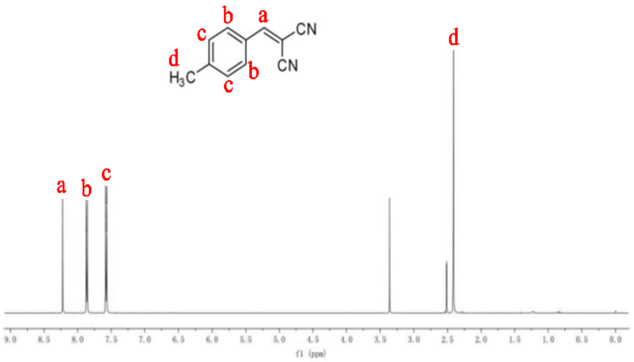

Fig. S12. ^{1}H NMR spectra of (4-methylbenzylidene) malononitrile.

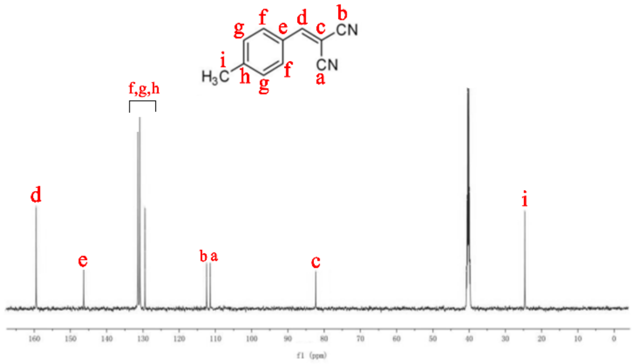

Fig. S13. ^{13}C NMR spectra of (4-methylbenzylidene) malononitrile.

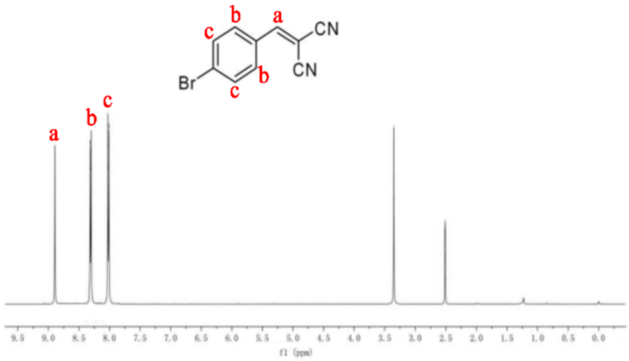

Fig. S14. ^{1}H NMR spectra of (4-Bromobenzylidene) malononitrile.

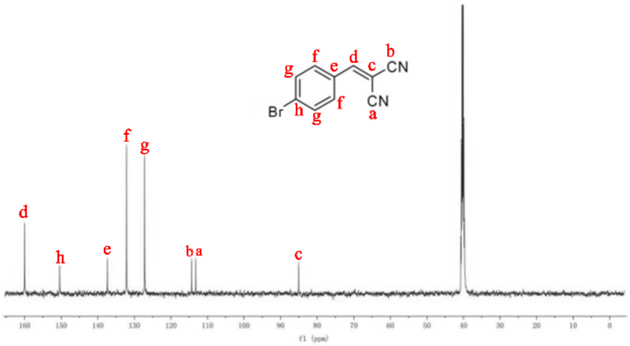

Fig. S15. ^{13}C NMR spectra of (4-Bromobenzylidene) malononitrile.

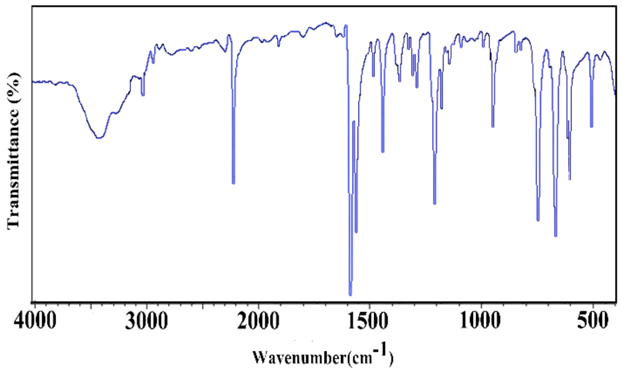

Fig. S16. FT-IR spectra of benzylidene malononitrile.

Table S2. Results of Knoevenagel condensation reaction of benzyl alcohol in the presence of catalyst **1**.

Entry	Amount of loading (%)	Conv
1	7.12	52
2	10.57	87
3	13.45	100

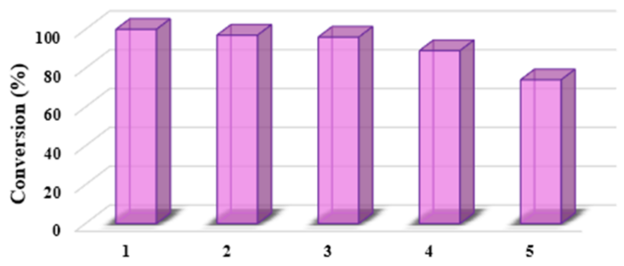

Fig. S17. The effect of recycling of HKUST-ED on the Knoevenagel condensation of benzaldehyde with malononitrile.

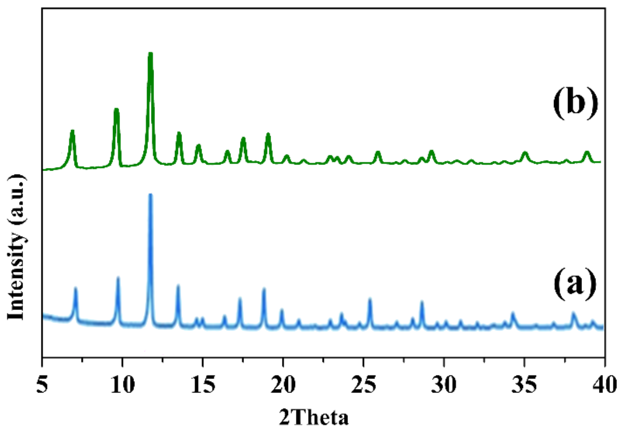

Fig. S18. XRD patterns of HKUST-ED (a) before and (b) after using as catalyst.

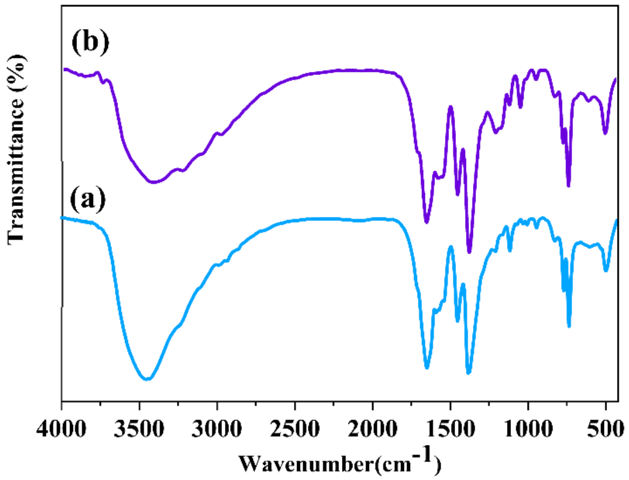

Fig. S19. FT-IR spectra of HKUST-ED (a) before and (b) after using as catalyst.
